# Genomic and physiological analyses of the zebrafish atrioventricular canal reveal molecular building blocks of the secondary pacemaker region

**DOI:** 10.1007/s00018-021-03939-y

**Published:** 2021-09-23

**Authors:** Karim Abu Nahia, Maciej Migdał, T. Alexander Quinn, Kar-Lai Poon, Maciej Łapiński, Agata Sulej, Jiandong Liu, Shamba S. Mondal, Michał Pawlak, Łukasz Bugajski, Katarzyna Piwocka, Thomas Brand, Peter Kohl, Vladimir Korzh, Cecilia Winata

**Affiliations:** 1 International Institute of Molecular and Cell Biology in Warsaw, Warsaw, Poland; 2 Institute of Molecular and Cell Biology, 61 Biopolis Dr, Singapore , Singapore; 3 Developmental Dynamics, National Heart and Lung Institute, Imperial College London, London, UK; 4 Department of Physiology and Biophysics, Dalhousie University, Halifax, Nova Scotia, Canada; 5 McAllister Heart Institute, University of North Carolina, Chapel Hill, USA; 6 Nencki Institute of Experimental Biology, Warsaw, Poland; 7 Institute for Experimental Cardiovascular Medicine, University Heart Centre, Faculty of Medicine, and Faculty of Engineering, University of Freiburg, Freiburg im Breisgau, Germany

**Keywords:** Atrioventricular canal, Atrioventricular node, Cardiac valve, Cardiac pacemaker, Zebrafish, RNA-seq

## Abstract

The atrioventricular canal (AVC) is the site where key structures responsible for functional division between heart regions are established, most importantly, the atrioventricular (AV) conduction system and cardiac valves. To elucidate the mechanism underlying AVC development and function, we utilized transgenic zebrafish line *sqet31Et* expressing EGFP in the AVC to isolate this cell population and profile its transcriptome at 48 and 72 hpf. The zebrafish AVC transcriptome exhibits hallmarks of mammalian AV node, including the expression of genes implicated in its development and those encoding connexins forming low conductance gap junctions. Transcriptome analysis uncovered protein-coding and noncoding transcripts enriched in AVC, which have not been previously associated with this structure, as well as dynamic expression of epithelial-to-mesenchymal transition markers and components of TGF-β, Notch, and Wnt signaling pathways likely reflecting ongoing AVC and valve development. Using transgenic line *Tg(myl7:mermaid)* encoding voltage-sensitive fluorescent protein, we show that abolishing the pacemaker-containing sinoatrial ring (SAR) through Isl1 loss of function resulted in spontaneous activation in the AVC region, suggesting that it possesses inherent automaticity although insufficient to replace the SAR. The SAR and AVC transcriptomes express partially overlapping species of ion channels and gap junction proteins, reflecting their distinct roles. Besides identifying conserved aspects between zebrafish and mammalian conduction systems, our results established molecular hallmarks of the developing AVC which underlies its role in structural and electrophysiological separation between heart chambers. This data constitutes a valuable resource for studying AVC development and function, and identification of novel candidate genes implicated in these processes.

**Supplementary Information:**

The online version contains supplementary material available at 10.1007/s00018-021-03939-y.

## Introduction

The atrioventricular canal (AVC) gives rise to the AV node, which constitutes part of the cardiac conduction system (CCS) responsible for generating and transmitting electrical impulses necessary for coordinated heart contraction [1, 2]. In the mammalian heart, the AV node can be found within the interatrial septum, at the AV junction [3]. Electrical impulses originating from the sinoatrial (SA) node are delayed by a fraction of a second in the AV node before being propagated further, ensuring consecutive contractions of the atrium and ventricle [4]. The AV node is often referred to as a secondary pacemaker as it possesses intrinsic automaticity, rendering it a potential arrhythmogenic source in cases where weakened or abnormal impulses from the SA node are not able to override it [3, 5]. In the mammalian embryonic heart, the AV myocardium forms a slow-conducting ring separating atrial and ventricular myocardium—a property retained in the AV node of the adult heart [2]. These cells express *Bmp4, Tbx2,* and *Tbx3,* which suppress the genetic program specifying fast-conducting working cardiomyocytes [6–8]. The electrophysiological properties of the AV node are determined mainly by the electrical coupling between its cells mediated by gap junctions. Connexins form gap junctions by either homogeneous or heterogeneous pairings, resulting in a different range of conductivity [9, 10]. In the mammalian heart, CX30.2 and CX45 form low or ultralow conductance gap junctions and are enriched in AV pacemaker cells [11, 12].

In zebrafish, the conductance delay between atrium and ventricle could be observed from 36 hpf [13]. Notch1b and Neuregulin expressed in the endocardium are required for the development of this conduction delay [14]. Zebrafish orthologs of *Tbx2* and *Tbx3* are expressed in the region corresponding to the mammalian AVC [15]; however, the molecular profile of this structure and its homologous function to the mammalian AV node is still poorly characterized. Islet1 (Isl1) [16, 17] is known to play a role in the development of the primary pacemaker, the SA node, as its deficiency causes cardiac arrhythmia [15, 18]. Isl1-positive cells in the sinoatrial region (SAR) of adult zebrafish co-express *hcn4*, which encodes the hyperpolarization-activated channel responsible for generating the pacemaker current (I_f_) [19]. In the AVC of adult zebrafish, a small group of *hcn4*-positive cells was found in the AV valves. However, in contrast to the SAR, Hcn4-positive cells in the AVC region were Isl1-negative [20]. The earliest expression of *hcn4* in zebrafish embryonic AVC was reported from 52 hpf [21], which suggests that it could potentially function as a secondary pacemaker. Electrical silencing of cells in the SAR region of the embryonic zebrafish heart using optogenetics abolished the heartbeat, suggesting that the activities of alternative pacemaker regions, such as the AVC, are not sufficient to drive heart contractions [22]. Interestingly, surgical isolation of the ventricle from the atrium led to the establishment of the AV region as the site of electrical activation origin, which revealed its pacemaking capacity although with a slower excitation rate [20].

Besides its role in cardiac conduction, mammalian AVC gives rise to the cardiac septa and valves, which provide structural division between the four heart chambers [2, 17]. In the zebrafish heart, AVC formation is initiated as early as 30 hpf when a constriction between the atrium and ventricle separates the two chambers [23]. As heart looping is initiated at 36 hpf, *bmp4* expression becomes restricted to the AVC myocardium [24], where it plays a role in the formation of cardiac jelly together with Has2. Valve development is initiated by the formation of endocardial cushions at two opposite sides of the AVC at 55 hpf [23]. By 60 hpf, some of these endocardial cells migrate into the cardiac jelly and undergo EMT, which provides the substrate for valve development [23]. Canonical Wnt signaling is required for zebrafish endocardial cushion formation [25, 26], although its precise mechanism is still unknown. In addition, Wnt signals originating from the endocardium induce myocardial *bmp4* and *tbx2b* expression necessary for patterning the AVC myocardium [27]. Similarly, *notch1b* and its ligand *dll4* are expressed in the AVC endocardium [24, 28] and are required for the formation of the endocardial cushion as well as AV conduction tissue [23, 29].

Given its role in the formation of major cardiac structures, disruptions to AVC development results in various forms of septal defects, and valve abnormalities leading to heart failure. In addition, defects of the AV node may lead to various degrees of AV block, which gives rise to cardiac arrhythmia [30]. However, our knowledge of genetic events responsible for the development and function of the AVC is still limited. One of the main challenges in studying the AVC is posed by their complex spatial anatomy and cellular heterogeneity [31]. Therefore, isolation of specific cell populations from ambient working myocardium and other surrounding tissues is challenging, limiting the identification of clear-cut molecular markers. Moreover, functional genetic studies of the AVC in higher vertebrates are impractical due to the early embryonic lethality caused by loss of function of essential genes [8, 32–35]. Thus, there is still a lack of reliable systems that can model the development, physiology, and pathology of the AVC.

Despite its significant evolutionary distance from humans, the zebrafish holds great potential to model human pathologies affecting the AVC and AV pacemaker function due to their conserved electrophysiological properties [36, 37]. An enhancer trap screen performed in zebrafish has generated a collection of transgenic lines expressing enhanced green fluorescent protein (EGFP) in different tissues or subdomains of the heart [36, 38]. The *sqet31Et* transgenic line expresses EGFP in a ring structure marking the AVC [38], which likely corresponds to slow conducting myocytes homologous to the mammalian AV node [13, 14, 38, 39]. We utilize the in vivo labeling of the AVC in *sqet31Et* to isolate cells making up this structure and perform detailed molecular characterization by transcriptome profiling at 48 hpf and 72 hpf, corresponding to the time of CCS and cardiac valve development. To better understand the physiology of the CCS in zebrafish, we characterized electrical conduction patterns between the SAR and AVC, and cross-compared the transcriptome profiles of both pacemaker regions. We show that the AVC gene expression profile exhibits hallmarks of the mammalian AV node and reflects ongoing biological processes implicated in valve development. The transgenic line *sqet33mi59BEt*, in which the enhancer trap was inserted close by the *fhf2* gene locus, expresses EGFP in the SAR [39]. Recently, we completed the analysis of the transcriptome of these cells [40]. Comparisons between the SAR and AVC transcriptomes revealed differences reflected in expression profiles of ion channels and connexins implicated in pacemaker function. A large number of AVC-enriched genes identified in this screen had human orthologs implicated in heart conditions related to cardiac conduction and valve/septal defects, suggesting the value of our transcriptome resource in identifying targets for further clinical investigations.

## Results

### Transgenic zebrafish line *sqet31Et* expresses EGFP in the zebrafish AVC

**Fig. 1:**
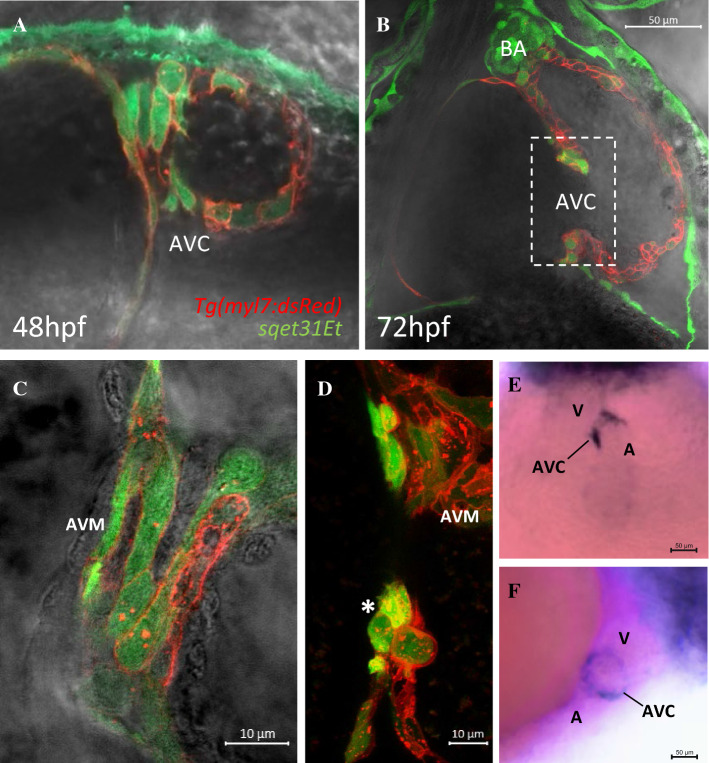
EGFP expression in the transgenic line *sqet31Et* defines cells of the AVC. **A**, **B** Confocal images showing the AVC region at 48 hpf and 72 hpf. Note the overlap between EGFP and mRFP signals, indicating extensive co-localization and confirming the largely myocardial nature of the EGFP expression domain in *sqet31Et*. **C**, **D** Close-up of the region marked in panel B at different focal planes showing the surface (**C**) and lumen (**D**) of the AVC. Note the group of three cuboidal cells protruding into the cardiac lumen at the location corresponding to the developing cardiac cushion (asterisk). **E**, **F** Whole-mount in situ hybridization of *egfp* in *sqet31Et* transgenic embryos at 72 hpf showing enrichment of EGFP expression in the AVC relative to the rest of the heart. *E* ventral, *D* lateral view, *A* atrium, *V* ventricle, *AVC* atrioventricular canal, *BA* bulbus arteriosus, *AVM* atrioventricular myocardium

The *sqet31Et* transgenic line carries the Tol2 transposon containing EGFP gene driven by a 460-bp basic promoter from the zebrafish *krt4* gene inserted in repetitive DNA region, which hinders mapping of the insertion site. The generation and detailed characterization of the line expressing EGFP in the bulbus arteriosus (BA) and AVC at high level and some myocardial cells at low level were described previously [38, 41]. To better visualize the GFP-expressing structure in *sqet31Et*, we performed high resolution imaging at 48 hpf and 72 hpf (Fig. 1A–D). We crossed *sqet31Et* with *Tg(myl7:mRFP)* that expresses membrane-bound RFP (mRFP) in cardiomyocytes. Confocal imaging of the AVC region revealed that at the surface of the AVC, the EGFP and mRFP expression overlapped, confirming the myocardial nature of the EGFP-expressing cells (Fig. 1C). At 72 hpf, two additional groups of ~ 3 cuboidal-shaped cells were detected at the deeper layer facing the cardiac lumen (Fig. 1D). These cells appear to be a part of the characteristic protrusion into the cardiac lumen most likely representing the developing AV cushion. Based on their location between the endocardium and myocardium, these cells have been previously defined as constituents of the non-chamber valve tissue [38, 39]. An additional EGFP expression domain detected previously [38] was observed at the BA from 72 hpf (Fig. 1B). The EGFP expressing domain in the developing heart of the *sqet31Et* transgenic line thus consists largely of AVC myocardium, with another expression domain in the BA, the latter observed only at 72 hpf.

### Transcriptome profile of the AVC

**Fig. 2:**
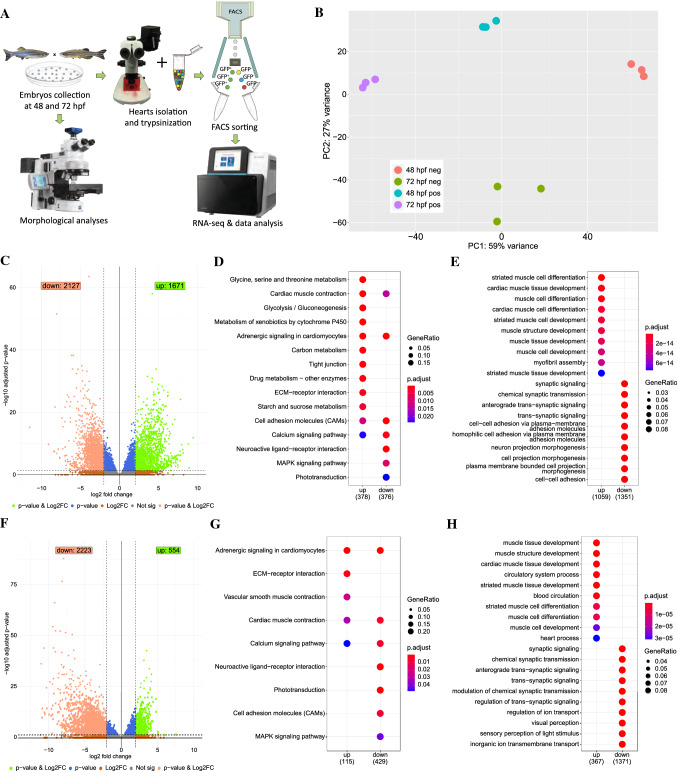
Transcriptome profiling of the GFP+ cells isolated from *sqet31Et.* **A** Scheme of experimental design. **B** Principal component analysis (PCA) on normalized RNA-seq data (regularized log) showing variance between three technical replicates of each sample as well as between samples. **C**, **F** Volcano plots showing genes differentially expressed between EGFP-positive and -negative cells at 48 hpf (**C**) and 72 hpf (**F**). DESeq2 was used to calculate log2FC and padj values. Green spots indicate genes considered as significant (padj < 0.05) with at least twofold change between groups (log2FC > 2). Accordingly, light red spots represent significant genes with log2FC smaller than -2. **D**, **G** KEGG pathway enrichment of differentially expressed genes at 48 hpf and 72 hpf. Enrichment analysis was performed on a gene list meeting the following criteria: log2FC > 2 or log2FC < -2 and padj < 0.05. Same criteria were used to perform biological process Gene Ontology terms enrichment on up- and down-regulated genes (**E**, **H**). Top ten terms for each enrichment analysis are shown

To characterize the molecular profile of the GFP + cell population in *sqet31Et,* we isolated these cells using fluorescence-activated cell sorting (FACS) at 48 hpf and 72 hpf (Fig. 2A) and profiled their transcriptome by RNA-seq. The rest of the heart cells, which did not express EGFP, were also collected (GFP−). Average sequencing reads mapping to the *egfp* sequence were considerably higher in GFP+ compared to GFP− samples, confirming the high representation of the EGFP-expressing cell population in the GFP+ samples (S1 Figure B). Principal component analysis (PCA) revealed tight clustering of replicates and clear separation between samples of different developmental stages (Fig. 2B).

To identify genes that are enriched in the AVC compared to the rest of the heart, we performed differential expression analysis between the GFP+ and GFP− fractions. In both developmental stages, a total of 3798 and 2777 genes were differentially expressed at 48 hpf and 72 hpf, respectively (absolute log2FC > 2, padj < 0.05), of which 1492 were common for both stages (Fig. 2C, F, S2 Table). GO and KEGG pathway enrichment analyses at 48 hpf revealed that the set of genes overexpressed in GFP+ compared to GFP− cells (absolute log2FC > 2, padj < 0.05; “AVC-enriched genes”) was overrepresented for functional terms related to cardiac muscle development and function (“cardiac muscle contraction”, “adrenergic signaling in cardiomyocytes”, “cardiac muscle development”, “cardiac muscle differentiation”, and “calcium signaling pathway”), in line with the myocardial identity of the GFP+ fraction (Fig. 2D, E, S3 Table). On the other hand, functional terms related to cell–cell adhesion ("cell adhesion molecules", "cell–cell adhesion") were overrepresented among transcripts overexpressed in the GFP- cell population. At 72 hpf, similar functional terms were overrepresented with the addition of “vascular smooth muscle contraction” term (Fig. 2G, H, S3 Table), which likely corresponded to the initiation of EGFP expression in the BA at this stage.

**Fig. 3:**
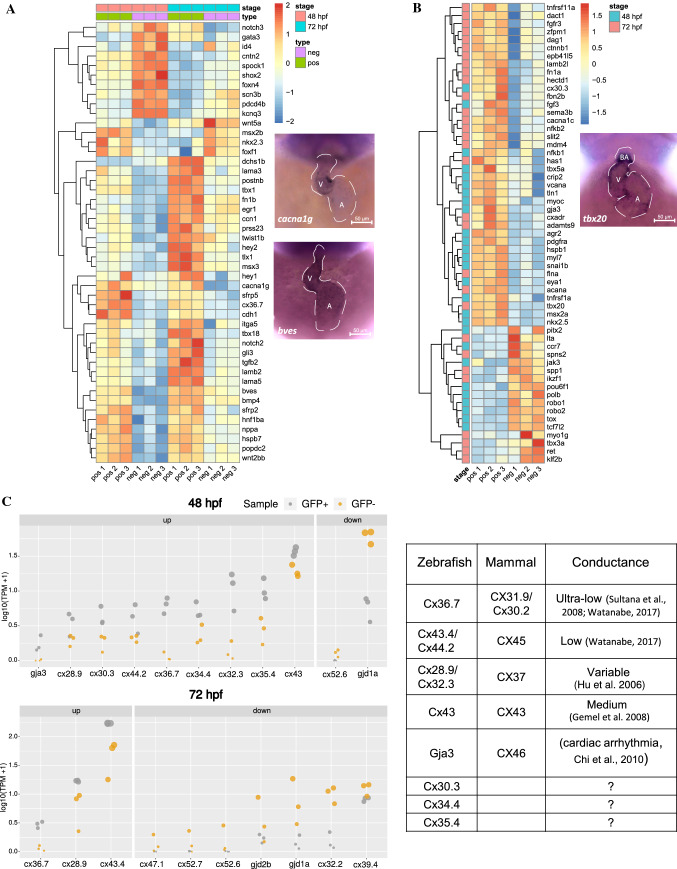
AVC gene signatures are enriched in *sqet31Et* EGFP-expressing cells. Signatures were retrieved from MGI and ZFIN databases and used to identify molecular markers associated with AVC expressed in the studied dataset (padj < 0.05). **A** Heatmap depicts the dynamic of changes of known molecular AVC signatures that are in common across the developmental stage. **B** AVC markers uniquely expressed in either 48 hpf or 72 hpf stage. **C** Expression (in log10(TPM + 1)) of genes encoding connexins, components of gap junctions which confer conductance properties between cells, in GFP+ and GFP− cells at 48 hpf and 72 hpf. Mammalian homologs of each connexin gene and their known conductance properties are described in the accompanying table

To assess whether GFP+ fraction contained AVC cells, we explored the presence of known markers of AVC in our dataset. We established a set of AVC marker genes for zebrafish and mammals by retrieving genes annotated with the term “atrioventricular canal” from the ZFIN (http://zfin.org/) and MGI [42] gene expression databases. Intersection of these known markers with the AVC-enriched transcriptome returned 46 and 58 genes in common, which were enriched in GFP+ cells at both 48 hpf and 72 hpf, respectively (Fig. 3A, B; S4 Table). Notably, several of these genes were known to be expressed specifically in zebrafish AVC myocardium, including *bmp4* [24], *wnt2bb* [43], *snai1b* [44], *hey2* [45], and *hnf1ba* [13]. On the other hand, 27 genes which were enriched in the GFP- population overlapped known AVC markers at either or both developmental stages. These included *id4* and *notch1b* reported to be expressed in the endocardium [29, 46], which suggests that the GFP+ cells in *sqet31Et* are less likely to be endocardial.

Besides protein-coding genes, we found 108 and 19 transcripts defined as long intergenic noncoding RNAs (lincRNAs) according to the ENSEMBL database which were overexpressed in GFP+ cells at 48 hpf and 72 hpf stages, respectively (padj < 0.05; S5 Table). Among the lncRNAs overexpressed at 48hpf is the lincRNA ALIEN (*linc.alien*, ENSDARG00000097567) and *si:ch211-265g21.1*. The mammalian ortholog of *linc.alien* is known to be expressed in cardiovascular progenitors, and its function in cardiac lineage specification was demonstrated in both mammals and zebrafish [47]. The lncRNA *si:ch211-265g21.1* was previously reported to be expressed in the embryonic zebrafish heart [48]. Additionally, MALAT-1 (*malat1*) which was reported to be expressed in heart [49] was found to be differentially expressed between 72 and 48 hpf. The rest of the lincRNAs in our list were mostly uncharacterized, which suggests the value of our transcriptome resources for discovering novel factors contributing to AVC development.

Besides the known AVC markers, the AVC-enriched transcriptome consisted of many other transcripts which were not previously associated with AVC or heart processes. To further validate the AVC-enriched transcriptome, we performed WISH on eight selected AVC-enriched transcripts with no previous heart expression reported. All of these were expressed in the AVC except for one, *si:dkey-57k2.6*, which is expressed only in the BA (S2 Figure). Another transcript, *si:dkey-164f24.2*, was expressed in the whole heart including the AVC (S2 Figure). Therefore, utilizing the *sqet31Et* transgenic line to specifically enrich for the GFP+ cell population, our RNA-seq analysis revealed the transcriptome representing the AVC, with contribution from the BA at 72 hpf.

To further discriminate the BA-associated genes from the AVC in the 72 hpf dataset, we intersected our data with a previously reported BA transcriptome of the adult zebrafish [100]. We found 56 out of 59 known BA-expressed genes enriched in GFP+ cells at 72 hpf (S6 Table). Unlike the AVC myocardium, the BA is mainly composed of smooth muscle [50]. Accordingly, in the 72 hpf dataset, we observed the enrichment of transcripts encoding smooth muscle light chain kinase (*mylka*), elastin B (*elnb*) and elastin A (*elna*) which is known to promote the differentiation of smooth muscle cell [51]*,* as well as *ltbp3* and *fbln5* implicated in maturation and function of elastin [51–53] (S6 Figure). Therefore, although the 72 hpf GFP+ transcriptome contained genes expressed in the BA in addition to AVC, their largely distinct tissue composition, as well as the availability of BA transcriptome data [54], allowed us to distinguish between them. On the other hand, the expression of EGFP in the AVC and BA of the *sqet31Et* transgenic line adds to a list of common markers of these cell lineages.

### AVC gene expression profile shows signatures of AV pacemaker

A hallmark feature of mammalian AVC myocardium is slow conduction, a property conferred by the composition of gap junctions between cells [9, 10]. Among the transcripts enriched in the GFP+ cell population, we found ten encoding various connexins. These consisted of *cx36.7* (ortholog of human CX31.9 and murine Cx30.2 [55, 56]) and *cx43.4/cx44.2* (ortholog of human CX45 [56]), whose mammalian orthologs form low or ultralow conductance gap junctions in the AV node [11, 12, 57]. These were enriched in the GFP+ population at both developmental stages, while other connexin transcripts were enriched only at 48 hpf (padj < 0.05). The latter group consisted of *cx28.9/cx32.3* (ortholog of human CX37 [56]), *cx43*, and *gja3/cx46* (Fig. 3C; S7 Table). Among those with the highest fold change between the GFP + and GFP- cell populations were *cx36.7*, *gja3/cx46*, and *cx32.3* at 48 hpf and *cx36.7* at 72 hpf. Loss of function of mammalian CX46 leads to cardiac conduction disorders, while the loss of *gja3/cx46* in the zebrafish mutant *dco* causes defects in heart morphology and ventricular conduction pattern [58]. The enrichment of genes encoding connexins forming low conductance gap junctions likely reflects the conduction delaying property of the AVC region. Besides those known for their role in cardiac conduction, transcripts encoding other members of the connexin family [Cx30.3 (CX30), Cx34.4 (CX30.3) and Cx35.4 (CX31)] were also enriched in the GFP+ cell population. These have not been previously implicated in heart or pacemaker function and are candidates for further investigation.

Besides delaying electrical conduction between atrium and ventricle, the AV node also possess intrinsic pacemaker activity [3, 5]. To determine whether this feature is conserved in the zebrafish, we searched amongst the GFP+ gene list for those known to be expressed in the AV node or associated with pacemaker development and function (S8 Table; [9, 59, 60]). Confirming previous reports [21], *hcn4* expression was observed in GFP+ cell population at both 48 hpf and 72 hpf (S3 Figure; S8 Table). In addition, genes encoding zebrafish orthologs of Tbx18, Shox2, and Tbx2/3 [61–63] were expressed in the GFP+ population (S3 Figure, S8 Table). In mammalian CCS, Tbx2/3 are known to repress the expression of the chamber-specific Cx40 [33, 61]. In agreement with this, *gja5a/b* (the zebrafish ortholog of CX40) was not AVC enriched. It has been shown that *nkx2.5* is expressed in all myocardium, but slightly higher in the AV conduction system [59]. Similarly, *nkx2.5* was enriched in GFP+ cells compared to GFP- at 48 hpf (S3 Figure, S8 Table). Taken together, the transcriptome of AVC myocardium reveals conserved features to that of the mammalian AV node in terms of expression of genes linked to slow conductivity, automaticity, and molecular mechanism for AV conduction system development.

### Defect of the primary SAR pacemaker reveals spontaneous activity of the AVC

**Fig. 4:**
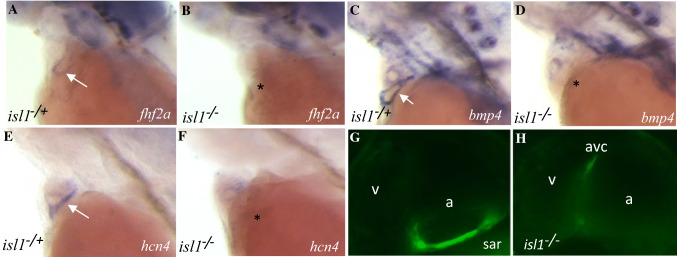
The absence of the pacemaker ring in *isl1* mutant causes the loss of expression of *fhf2, hcn4, bmp4*. **A**–**H** Expression pattern (labeled with arrow) of *fhf2a* (**A**, **B**), *bmp4* (**C**, **D**), *hcn4* (**E**, **F**) and EGFP (**G**, **H**) in *isl1* siblings and mutants. (**A**–**F**) WISH, (**G**, **H**) confocal microscopy of CCS in the sibling and *isl1* mutant

The expression of *hcn4* and other AV node markers in the zebrafish AVC led us to question whether it possesses inherent pacemaking activity as does its mammalian counterpart. We utilized *isl1* K88X mutant (*isl1*^sa29^), which exhibits a defective SAR pacemaker function manifested as sinus pauses and bradycardia [15, 18]. Apart from lacking the expression of *fhf2a* (Fig. 4A, B), *bmp4* (Fig. 4C, D), and *hcn4* (Fig. 4E, F) in the sinus venosus, *isl1*^*−/−*^ was devoid of EGFP-positive cells at the SAR, but not the AVC, as shown by analysis of *sqet33mi59BEt* [39] (Fig. 4G, H). Hence, we confirmed that the reduced number of cardiomyocytes at the venous pole in *isl1*^*−/−*^ observed previously [18] resulted from the absence of the pacemaker cells containing SAR. It is worth noting that *isl1*^*−/−*^ is the only vertebrate mutant that shows a complete lack of pacemaker SAR cells.

**Fig. 5:**
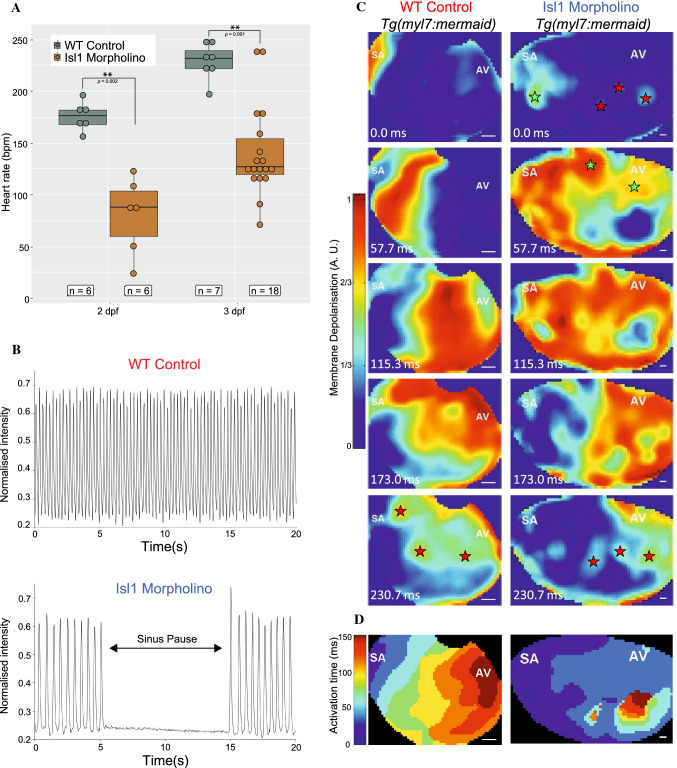
Effects of morpholino knockdown of *isl1* on electrical activity of the atria in zebrafish larvae. **A** Heart rate at 48 hpf (left) and 72 hpf (right) in WT control (grey) and *isl1* morphant (brown) zebrafish, showing slowed heart rate in *isl1* morphants (Wilcoxon rank sum test with continuity correction, asterisks indicate *p value* ≤ 0.01). **B** Videographic analysis of the heartbeat in WT control (top) and *isl1* morphant (bottom) 48 hpf zebrafish, showing slowed heart rate and sinus pauses (during the period indicated by the arrow) in the *isl1* morphant. **C** Sequence of video frames showing electrical activation of the atria in WT control (left) and *isl1* morphant (right) 48 hpf *Tg*(*myl7:mermaid*) zebrafish, showing normal activation (from the sinoatrial node region [SA] to the atrioventricular [AV] junction) and sites of latest activation/repolarisation (indicated by red stars) in the WT control and abnormal activation in the *isl1* morphant (sites of early/ectopic activation in the morphant zebrafish indicated by green stars). **D** Isochronal activation map of the atria in WT control (left) and *isl1* morphant (right) 48 hpf zebrafish derived from the video represented in (**C**)

Despite the complete absence of SAR pacemaker, Isl1-deficient hearts contract, albeit inefficiently and irregularly, with long pauses (Fig. 5A, B, Supplementary movies 1 and 2). This suggests the existence of alternative origins of automaticity that triggers the initiation of cardiac contractions. To investigate whether the AVC could generate electrical impulses independent of the SAR, the Isl1 antisense morpholino [64] was injected into 1–4 cell stage zebrafish embryos expressing the genetically encoded voltage-sensitive fluorescent protein Mermaid, *Tg(myl7:mermaid)*. This allowed direct observation of cardiac electrical conduction patterns. Similar to *isl1* mutants, about 66% of Isl1 morphants showed sinus pauses (6 of 9 morphants vs 4 of 6 mutants), and all showed bradycardia. The heart rate of Isl1 morphants was 80.2 ± 15 beats per minute (bpm) at 48 hpf (mean ± SEM., *n* = 6) and 141.2 ± 10.3 bpm at 72 hpf, (*n* = 6), which is significantly lower than that in controls (176 ± 5.7 bpm at 48 hpf, (*n* = 7) and 229 ± 6.6 bpm at 72 hpf, (*n* = 18)) (Fig. 5A). Increased variability in heartbeat duration was noted in the Isl1 morphants as well.

In control hearts, the excitation wave front traveled uniformly across the atrium from the SAR toward the AVC (Supplementary movie 1; Fig. 5C, D). In contrast, in Isl1 morphants, the origin of the atrial excitation wave, although still predominantly from the SAR region, was more disperse and less coordinated, presumably driven by secondary pacemakers and automaticity of atrial cardiomyocytes (Supplementary movie 2; Fig. 5C, D). During the sinus pause, several stationary centers of automaticity distributed from posterior to anterior were detected, with the SAR region showing the highest activity. Interestingly, the location associated with AVC became more active near the end of the atrial contraction (Supplementary movie 2, Fig. 5C, D, asterisk), when triggering the subsequent phase of the excitation in the ventricle. The activity of the AVC location became even more pronounced during the periods of sinus pause. The loss of coordinated excitation wave and conduction in Isl1 deficient embryos suggests that the SAR is the primary pacemaker required for coordination of excitation wave. However, coordinated ventricular excitation can be induced by the electrical activity of the AVC with lower inherent pacemaker rate, in particular when no wave of excitation from the SAR drives the heartbeat.

### Comparison between the AVC and SAR transcriptomes reflect distinct electrophysiological properties

The differences in the inherent activation rates of the SAR and AVC regions of the zebrafish heart led us to question the molecular nature underlying their distinct properties. We compared the transcriptome of the AVC with that of the SAR [40] to identify differentially enriched genes. Intersection between the two transcriptomes obtained a total of 1516 AVC-unique, 701 SAR-unique, and 450 common genes (S9 Table). Interestingly, while *hcn4* was expressed in both SAR and AVC, its expression was enriched compared to the rest of the heart in the SAR but not the AVC. This may reflect the role of SAR as the dominant pacemaker (S9 Table). Several other transcripts encoding various ion channels were enriched in both SAR and AVC, notably, the T-type calcium channel Cacna1g, which is necessary for mammalian pacemaker activity in both SA and AV nodes [65]. Genes enriched only in AVC include *trpm4* encoding a Ca^2+^-activated nonselective cation channel [66], which is implicated in human progressive familial heart block type I characterized by cardiac conduction blockage downstream of the AV node [67]. Another notable example is *cacna1c*, whose human ortholog is associated with the Wolff–Parkinson–White syndrome, a condition affecting the AV conduction system [68, 69]. Other genes, including *kcnq1.1*, *kcne4*, and *atp1b1a*, possess human orthologs associated with the maintenance of QT interval [70–72].

Despite having some common properties, the SA node serves a primarily pacemaking function, while the AV node is mainly specialized to delay electrical propagation between the atrial and ventricular chambers [9]. Therefore, while both regions express partially overlapping, mostly low-conducting gap junction proteins [9, 73], the AV node is particularly enriched in Cx30.2 and Cx45 [33, 74]. Accordingly, *cx36.7,* the zebrafish paralog of *Cx30.2*, was enriched in the AVC but not the SAR (S9 Table). On the other hand, *cx43.4*, a paralog of *Cx45,* was enriched in both the SAR and AVC (S9 Table). Low electrical coupling is also a necessary property within the definitive pacemaker cells of the SA node to prevent inhibitory interference from the surrounding working myocardium, which is more hyperpolarized [9]. Collectively, the overall differences in ion channel, cell adhesion, and extracellular matrix composition enriched in the SAR and AVC likely underlie their distinct electrophysiological properties.

### Developmental signaling pathways dynamics suggests signaling pathways implicated in valve formation

**Fig. 6:**
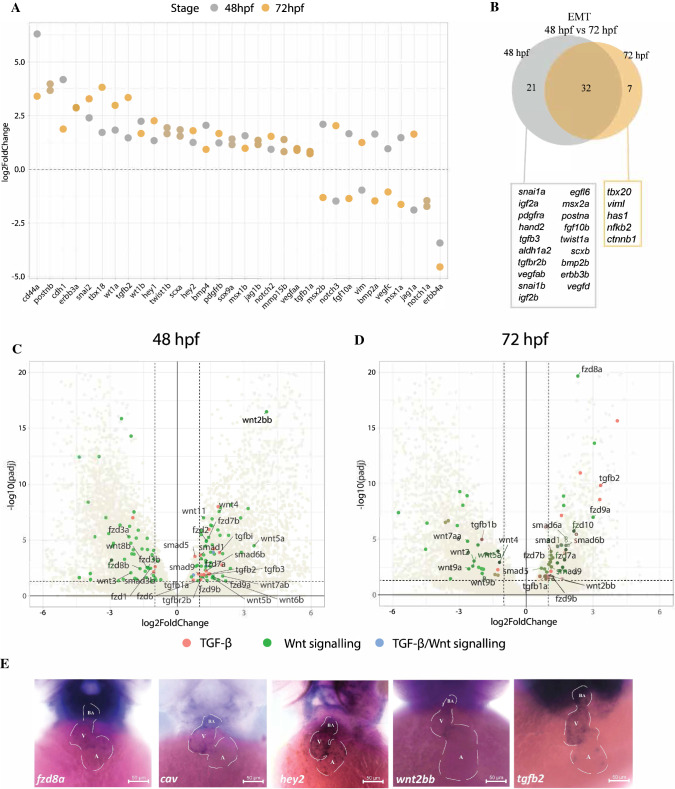
The transcripts of genes involved in EMT and valve development are enriched in the AVC. **A** AVC enrichment (expressed in log2-fold change between GFP + and GFP− cells, padj < 0.05) of genes known to regulate EMT at both 48 hpf and 72 hpf stages. **B** Overlap of EMT-regulating genes enriched in GFP+ cells at both stages. **C**, **D** Volcano plot showing enrichment of components of the TGF-β and Wnt signaling pathways in AVC at both 48 hpf and 72 hpf stages. **E** Whole mount in situ hybridization of several AVC-enriched genes of the TGF-β and Wnt pathway components

Besides hosting the AV node, the AVC is also the site where cardiac valve formation is initiated. We identified transcripts encoding genes involved in the EMT process enriched in the GFP+ cell population (Fig. 6A, B; S10 Table). Members of the TGF-β signaling pathway were enriched in GFP+ cells at both developmental stages (*tgfb2*, *tgfb1a, smad1, smad6b, and smad9*) or specific to either stage (*tgfb3, tgfbr2b,* and *smad6a)* (Fig. 6C). This is in line with the observation in mammalian endocardial cushion formation, where various TGF-β ligands are expressed in different cell populations of the AVC [75]. Notch signaling activity in the AVC endocardium is necessary for inducing EMT [76, 77] and transcripts encoding its key components were enriched in GFP + cells at both developmental stages (S2 Table). These include *jag1b*, *hey1*, and *hey2,* and *prss23*, implicated in AV valve formation [78]. In contrast, valve endocardial markers *notch1b* and its ligand *dll4* [23, 24, 28] were not enriched in GFP+ cells, which further supports the non-endocardial identity of this population.

Components of the canonical Wnt signaling have been reported to be expressed in different cell layers of the mammalian AVC, including *Wnt2*, *Fzd2*, and *Lef1* in the cushion mesenchyme and *Wnt4* and *Wnt9b* in endocardial endothelium [79]. In the zebrafish AVC, *wnt2bb* was enriched in GFP+ cells at both stages, while *wnt7ab*, *wnt6b*, *wnt5a/b*, and *wnt4* were enriched only at 48 hpf (Fig. 6C). Genes encoding Wnt signaling receptors *fzd7a/b*, *fzd9a/b, fzd2, fzd6, fzd6*, *fzd8a,* and *fzd10* were enriched in both or either stages (Fig. 6C), whereas *dkk1a/b* and *dkk2* encoding Wnt antagonists were enriched at 48 hpf. To visualize Wnt signaling activity in the AVC region, we crossed the *sqet31Et* transgenic line with the Wnt reporter line *Tg(7* × *TCF-Xla.Sia:NLS-mCherry)* [80]. In agreement with previous reports of the presence of Wnt signaling in the AVC myocardium [27, 81], we observed that both the non-EGFP-expressing endocardium and EGFP-expressing cells had Wnt signaling activity at both developmental stages (S4 Figure). Comparing between the two developmental stages, a total of 5877 genes were differentially expressed at 72 hpf compared to 48 hpf (padj < 0.05; − 1 < log2FC > 1; S5 Figure, S11 Table). Notably, GO terms related to TGF-β, Wnt, and Notch signaling pathways were overrepresented at 72 hpf (S12 Table). Members of these signaling pathways exhibited dynamic expression between 48 and 72 hpf (S5 Figure; S13 Table). Collectively, our observations uncover the dynamic expression of various components of the TGF-β, canonical Wnt, and Notch signaling pathways in the AVC myocardium, which likely reflects their role in the ongoing AVC patterning and valve development. Although these signaling pathways themselves were known to be implicated in AVC and valve development, components of these pathways which were directly involved in this process were largely unknown. Whole mount in situ hybridization confirmed the expression of some of these components in the AVC region (Fig. 6E). The identification of these factors would allow deeper understanding of the molecular networks governing AVC formation and function.

### AVC-enriched genes are associated with human congenital heart defects related to CCS, valves and septa

We identified human orthologs of AVC-enriched genes and interrogated them for any association with clinical phenotypes related to ClinVar terms: “arrhythmia”, “AV block”, “long QT syndrome”, and “conduction”. Our analysis revealed a total of 91 and 60 unique genes associated with these four ClinVar terms at 48 hpf and 72 hpf stages, respectively (S14 Table). Specifically, disease conditions represented by these terms included general forms of cardiac arrhythmia such as atrial fibrillation, sick sinus syndrome, abnormal QT interval, and Brugada syndrome, as well as those conditions specifically associated with defects of the AV conduction system or downstream effects such as heart block [82], Wolff–Parkinson–White pattern [69], and supraventricular tachycardia [83]. The latter group included *trpm4* and *cacna1c,* which were enriched in both SAR and AVC, as well as *mybpc3, smyhc2, hrc, dspa, myh7l, zgc:86709, lmna, snta1*, and *ttn.2*.

As the AVC is also the site where endocardial cushion and valve develop, we expected to find associations between AVC-enriched genes and human valve and septal defects. We searched human orthologs of AVC-enriched genes for overlap with ClinVar terms containing “tricuspid valve”, “AV valve”, “mitral valve”, and “valve in general”. In total, 115 and 93 unique genes were associated with these terms at 48 hpf and 72 hpf, respectively (S15 Table). In addition, 66 and 55 unique genes were associated with the term “septal defect” at each respective stages (S16 Table). In the adult human heart, AV node is embedded into the interatrial septum [3]. Given that the endocardial cushions are involved in the formation of the AV valves and septa, defects of interatrial septum could be linked to defects in cardiac conduction. In fact, a number of genes were commonly associated with ClinVar terms “cardiac conduction” and “valve” (S16 Table). For example, *tbx5a*, whose human ortholog TBX5 causes the Holt–Oram syndrome characterized by congenital heart malformation due to variable atrial and ventricular septal defects as well as heart conduction defects [74, 84]. Another notable example is *smyhc2*, whose human ortholog MYH6 is associated with both atrial septal defect and sick sinus syndrome [85, 86]. Other examples include *cacna1c*, *ttn.2*, *snta1*, *lmna*, *dspa*, and *mybpc3.* The overlap of a large number of AVC-enriched genes with human heart conditions related to CCS and valve/septal defects suggests our transcriptomics data as a valuable resource for studying these diseases.

## Discussion

The AVC constitutes part of the CCS which serves as the site where the propagation of electrical impulses is delayed, allowing consecutive contraction of the atrium and ventricle. In addition, it is also the site where the heart valves develop. The study of the AVC is challenging due to the lack of specific molecular markers defining this region. Available data relied on methods based on histological sections [87], which lacks the ability to isolate specific cell types. Nevertheless, it is known that distinct structures of the AVC, such as the pacemaker cells and cardiac valve tissue, express unique combinations of marker genes which can be used to distinguish them. The transgenic line *sqet31Et* provides the necessary level of specificity, which allows the enrichment of AVC myocardial cells by FACS. A caveat of this approach remains in the fact that the EGFP expression pattern in the *sqet31Et* transgenic line is driven by the activity of a yet unknown enhancer [38], which prevented an accurate assessment of the homogeneity of cell populations expressing EGFP in this transgenic line and their precise identity. For now, it is challenging to identify this enhancer due to the insertion of the enhancer trap construct in genomic repeat regions. However, with the increasing availability of long read sequencing methods, it may be possible in the near future to map the insertion site and trace the identity of this enhancer. Nevertheless, the co-expression of EGFP in the AVC and BA and cell populations suggests a unifying regulatory principle governing the specification of different cell types spatiotemporally. Transcription factors such as Tbx3 are expressed in both AV node and outflow tract mesenchyme, suggesting a similarity of developmental mechanism [88]. This poses an interesting question on gene regulation by the regulatory element(s) driving the expression pattern in *sqet31Et* transgenic line.

Our transcriptome analyses revealed that the zebrafish AVC myocardium possesses hallmarks of the mammalian AV node. The AVC transcriptome is characterized by high expression of mRNA encoding low conductance connexins *cx36.7* and *cx43.4*, as well as the T-type calcium channel *cacna1g* and pacemaker hyperpolarization-activated channel *hcn4*. All these factors are known to define the AV node and pacemaker activity in the mammalian heart [11, 57, 65, 89, 90]. The conserved features also extend to the expression of transcripts encoding the core pacemaker transcriptional network consisting of Tbx2a/2b/3/18 and Shox2 transcription factors.

The existence of the CCS in zebrafish has been supported by optogenetic studies [20, 22] with other evidence suggesting that the endocardium and hemodynamic stimulation play an important role in its development [39, 46, 91]. The SA pacemaker has been relatively better characterized, and it has been shown that its activity depends on Isl1 [15]. Using the *sqet33mi59BEt* transgenic line, we show that the loss of Isl1 abolishes the SAR harboring the primary pacemaker activity. Analysis of electrical conduction patterns in *isl1* morphants revealed disorganized excitation, which still generally progressed from the SA to AV, but included sites of ectopic automaticity. This suggests that by driving primary pacemaker function, Isl1 acts to coordinate atrial activation. However, we cannot rule out the possibility that the *isl1* knockdown did not abolish its function completely. In either case, the lack of an organized atrial activation pattern affects overall cardiac contraction, indicating that the coordinated signaling from the SAR and its propagation play a crucial role in coordinating heart contraction. The increase in AVC activation during the pause in heart rhythm in Isl1-deficient embryos demonstrated that it possesses inherent automaticity, enabling it to independently excite when a weakened signal from the primary SAR pacemaker is not sufficient to drive heart contractions. This corroborates previous observations in adult zebrafish heart of spontaneous electrical activity at the AV region following surgical uncoupling of the ventricle from the atrium [20]. Comparison of the transcriptome profiles of the SAR and AVC, while revealing common markers of pacemaker activity such as *cacna1g* and *cx43.4*, also indicated differences that would affect electrical properties, such as the enrichment of distinct types of ion channel, gap junction, and extracellular matrix components. This further supports that, despite their shared ability to act as a pacemaker, the SAR and AVC performs different functions.

Mammalian AV node consists not only of definitive pacemaker cells, but also fibroblasts, macrophages, and ECM, which provide electrical insulation around the AV node [2, 30]. Electrical impulses from AV node are further propagated by the His/Purkinje fiber network, which link to the thick myocardial tissue throughout the whole ventricle [31]. In contrast, the two-chambered heart in most fishes resembles the mammalian embryonic heart tube, where electrical current is propagated from one end to the other by means of electrical coupling of cardiomyocytes without a specialized CCS [2]. Hearts of ectothermic animals contain no insulating fibrous structure, although the slow conducting muscles of the AVC is present [92]. Moreover, teleost hearts are not known to possess any defined Purkinje fiber network, and conduction function is served by the ventricular trabeculae, which form myocardial continuity between AVC and apex of the ventricle [93]. Therefore, it is reasonable to assume that CCS function could be served by an equally simplified structure, in which a subset of cardiomyocytes performs pacemaking function and at the same time express additional attributes, which enable it to slow down electrical propagation. Intriguingly, it was previously observed that cells of the embryonic SAR send processes into the AVC, which appeared as a network connecting the two structures [39]. It is therefore tempting to speculate that a previously uncharacterized structure or cell type may exist in the zebrafish, which facilitates fast conduction between the SAR and AVC.

Besides serving as part of the CCS, the AVC is also the site where the heart valves and septa develop. Accordingly, the AVC transcriptome was enriched for transcripts encoding regulators of EMT, which is a hallmark of endocardial cushion and valve development. In addition, components of major signaling pathways, including Wnt, Notch, and TGF-β, which were implicated in endocardial cushion and valve development [28, 94], were differentially expressed at 48 to 72 hpf. Canonical Wnt signaling is known to play multiple roles in valve development, including regulation of AVC maturation and establishment of its electrical properties upstream of Tbx3 [81]. In the adult zebrafish heart, a compact group of Hcn4-positive cells is embedded within the musculature of the AV valves [20]. The close association between the valve tissues and pacemaker cells is reflected in our transcriptome and adds to the heterogeneity of cell types present within this region. Currently, bulk RNA-seq approach does not allow us to distinguish between the various cell subpopulations, or to clearly demarcate the concurrent developmental processes within the AVC region. Analyses at the single-cell level in both embryonic and adult zebrafish hearts are ongoing which is expected to reveal the true cellular diversity of this structure and more accurately characterize the CCS organization in zebrafish heart.

## Conclusions

Collectively, our results establish that the zebrafish AVC possesses molecular and physiological hallmarks of a secondary pacemaker, similar to that of the mammalian AV node, in terms of automaticity, low conductance properties, and conserved expression of developmental genes. The partially overlapping expression profiles of genes encoding ion channels and connexins likely underlies the distinct conduction functions between the SAR and AVC. In addition, the dynamic expression of signaling pathways implicated in the ongoing valve development illustrates the role of the AVC in both electrophysiological as well as structural separation between the heart chambers. The AVC transcriptome data generated in this study will enrich our knowledge of molecular factors, including novel candidate genes and noncoding transcripts, implicated in cardiac conduction and valve development.

## Methods

### Zebrafish

Wild-type, *sqet31Et* and *sqet33mi59BEt* enhancer trap [38, 39], and other zebrafish lines used in this study: *Tg(myl7:mRFP)* [95]*,* Wnt reporter line *Tg(7xTCF-Xla.Siam:nlsmCherry)* [80], were maintained in the zebrafish facility of the International Institute of Molecular and Cell Biology in Warsaw (license no. PL14656251) in line with standard procedures and ethical guidelines. *Tg(myl7:mermaid)* was generated by injection of a *Tol2-myl7-mermaid* construct (kind gift of Yasushi Okamura), together with transposase RNA, into one- to two-cell stage AB zebrafish embryos, followed by screening for fluorescence progeny. The *isl1* K88X mutant (*isl1*^sa29^) and *Tg(myl7:mermaid)* were bred and maintained at the Harefield Heart Science Centre according to the Animals (Scientific Procedures) Act 1986. Embryos were raised in egg water at 28 °C, screened for a fluorescence signal in the heart and staged at 48 hpf and 72 hpf based on established morphological criteria [96]. The generation and characterization of the isl1 K88X mutant (isl1sa29) was previously described [97].

### Heart extraction and fluorescence-activated cell sorting (FACS)

To isolate the heart, embryos were anesthetized with Tricaine (0.16 mg/ml in egg water) and large-scale extraction was performed according to a previously published protocol, with minor adjustments [98]. GFP-expressing hearts were manually separated from remaining tissue under a fluorescence stereomicroscope and collected into 0.5 ml of EDM (L-15/10% FBS). Pools of 300–500 hearts were dissociated with Trypsin–EDTA solution (0.05%) as previously described [99]. A FACS Aria II cytometer (BD Biosciences, USA) was used to enrich GFP positive (fluorescent) and GFP negative (nonfluorescent) heart fractions. Gates for cell sorting were calibrated against dissociated hearts extracted from wild type zebrafish embryos at the respective developmental stages (48 hpf or 72 hpf). On average, FACS yielded 15–25% of GFP+ events of total singlet events (Supplementary Fig. 1A).

### RNA extraction

To obtain high-quality total RNA, cells were sorted directly to 500 µl TRIzol™ LS Reagent (Thermo Fisher Scientific, USA) followed by RNA purification and DNase I treatment by means of a Direct-zol™ kit (Zymo Research, USA) according to the manufacturer’s protocol. The Tapestation 2200 and High Sensitivity RNA ScreenTape assay (Agilent Technologies, USA) together with Quantus™ Fluorometer (Promega, USA) were used to assess quantity and quality of total RNA. The average RNA Integrity Number equivalent (RIN^e^) for samples used for downstream analysis was 8.7.

### Library preparation and sequencing

To obtain sequencing libraries, a two-step approach was applied. First, cDNA carrying full-length transcript information was synthesized with SMART-Seq® v4 Ultra® Low Input RNA Kit for Sequencing (TaKaRa Bio, Japan), followed by Nextera XT DNA Library Preparation Kit (Illumina, USA) according to the manufacturer's guidelines. As previously, Tapestation 2200 and dedicated High Sensitivity D5000 ScreenTape and High Sensitivity D1000 ScreenTape assays were used to validate final cDNA and sequencing libraries, respectively. Final libraries were quantified with KAPA Library Quantification Kit Illumina® Platforms (Kapa Biosystems, USA), followed by paired-end sequencing (2 × 75 bp) performed with Nextseq 500 (Illumina, USA). Libraries were sequenced in triplicate, where a single replicate consisted of GFP-positive and GFP-negative fractions for both developmental stages (48 hpf and 72 hpf), at an average depth of 47 million reads.

### Analysis of sequencing data

FastQC tool v. 0.11.8 [100] was used to assess the quality of obtained raw RNA-seq reads. Minor adapters contaminations were removed by Cutadapt v. 1.17 [101] and RNA-seq reads were mapped to the zebrafish reference genome (GRCz11) using Salmon tool v. 0.9.1 [102] resulting in an average of 75% mappability rate (S1 Figure C). Sequencing reads were further analyzed in R programming language v. 3.5.2 [103], whereas differentially expressed genes were identified by the DESeq2 package [104]. Principal component analysis was performed on normalized reads counts transformed to the log2 scale by *plotPCA* function from the same package. ClusterProfiler v. 3.17.3 [105] was used to calculate the enrichment of both biological processes of Gene Ontology terms as well as KEGG pathways. The *enrichGO* and *enrichKEGG* functions were used with default *pvalueCutoff* and *qvalueCutoff* parameters. The ggplot2 package [106] was utilized for plots generation. Discovery of lincRNAs was performed using Ensembl GRCz11 primary assembly (v. 103) and ZFIN.

### Confocal imaging

Embryos used for imaging were grown in egg water supplemented with 0.003% 1-phenyl 2-thiourea (PTU) at 24 hpf to prevent the formation of melanophores and pigmentation. Prior to imaging, embryos were anesthetized with 0.02% tricaine (MS-222; Sigma-Aldrich A5040), embedded in 1% low-melt agarose (Sigma, USA) in egg water, and mounted in a glass-bottom dish before imaging on an inverted confocal microscope (LSM800, Zeiss). Images were further processed with Imaris 8 software (Bitplane).

### Optical mapping of atrial excitation

To visualize excitation in the embryonic heart, a transgenic zebrafish line expressing the FRET-based voltage-sensitive fluorescent protein Mermaid [107] specifically in myocardial cells *Tg(myl7:mermaid)* was used and optical mapping was performed as described previously, with minor adjustments [108]. Injection of morpholino against *isl1* (5′-TTAATCTGCGTTACCTGATGTAGTC-3′) was performed as previously described [64]. Taking into account sample distributions, variances, and sample sizes we decided to apply a non-parametric test for both conditions (Wilcoxon rank sum test with continuity correction) which resulted in *p* value = 0.002165 (at 2 dpf) and *p* value = 0.001483 (at 3 dpf). Embryos were embedded in 1% low melting agarose on a 35 mm Petri dish and oriented ventral side up to the imaging plane. Embedded embryos were transferred to an imaging chamber (RC-29; Harvard Instruments, USA) with a heated platform (PH-6D; Harvard Instruments). The temperature was maintained at 28 °C by a temperature controller (TC-344B; Harvard Instruments). Images were obtained using an epifluorescence upright microscope (BX51WI; Olympus) and focusing module (BXFM; Olympus) with a 40X water immersion objective (LUMPLFLN 40XW; Olympus) and magnification changer (U-CA; Olympus). Fluorescence was excited using a blue light-emitting diode (CBT-90; Luminus, USA) passed through a 460 ± 5 nm bandpass filter (HQ460/10X; Chroma, USA). Fluorescence was collected with a 482 nm dichroic mirror (FF482-Di01; Semrock, USA). To obtain simultaneous images of FRET donor and acceptor signals, collected light was passed into an image splitter (OptoSplit II; Cairn Research, USA), split with a 552 nm dichroic mirror (FF552-Di02; Semrock), and passed through either a 500 ± 30 nm (HQ500/60 m-2p; Chroma) or 600 ± 37.5 nm (HQ600/75 m; Chroma) bandpass filter. Filtered emission was projected to two halves of a 16-bit, 128 × 128, 24 mm^2^ pixels, cooled electron multiplying charge-coupled device camera (Cascade: 128 + ; Photometrics, USA) and collected at 52 Hz with a ~ 19 ms exposure time. Images were processed and analyzed using custom routines in MATLAB (R2011b; MathWorks, USA). The ratio of FRET donor and acceptor signals was taken and spatially filtered using the pixelwise adaptive, linear, noise-removal Wiener method (‘wiener2’) with a 3 × 3 pixel window. The atrium was manually segmented and each pixel signal normalized through time. Activation time was measured as the point at which the rate of voltage upstroke was maximal.

### Electrophysiology

Micropipettes for electrocardiograph (ECG) measurement on whole zebrafish larvae were prepared by pulling fire-polished borosilicate glass capillaries (World Precision Instruments) using the Flaming/brown micropipette puller P-1000 (Sutter Instrument). The zebrafish larvae were mounted (laterally) in 1% low melting agarose in a glass dish and submerged in external buffer: 1 × egg water (0.6 g/L sea salt in reverse osmosis purified water). The micropipette was filled with internal buffer (174 mM NaCl, 2.1 mM KCL, 1.2 mM MgSO4.7H20, 1.8 mM Ca(NO3)2.4H2O, 15 mM HEPES, pH 7.2) and the tip was positioned right above the pericardial region of the zebrafish heart. The electrical signals from the zebrafish heart received were recorded by pCLAMP 10 software (Molecular Devices) after amplification via Multiclamp 700B amplifier (Molecular Devices) and digitization through Axon Digidata 1440A digitizer (Molecular Devices). Data were analysed with Clampfit 10 software (Molecular Devices).

### Whole mount in situ hybridization

For antisense probes generation, total RNA from 72 hpf embryos was extracted and reverse transcribed into cDNA with SuperScript IV Reverse Transcriptase (Thermo Fisher Scientific, USA). Obtained cDNA was used as a template for PCR. Purified PCR products were used as a template for in vitro transcription from the T7 promoter. Primers used are listed in S1 Table or reported previously [39]. Whole mount in situ hybridization (WISH) was performed as previously described, with minor adjustments [109]. Zebrafish embryos were grown in egg water containing PTU and fixed overnight at desired developmental stage in 4% paraformaldehyde in 1 × PBS (PFA/PBS). After sequential washes with 1 × PBT (50 ml 1 × PBS + 250 µl 20% Tween-20), embryos were digested for either 30 min (48 hpf) or 50 min (72 hpf) with 10 µg/ml proteinase K (Roche), washed with 1 × PBT, and fixed again for 1 h. PFA/PBS solution was discarded, and embryos were pre-hybridized overnight at 68 °C in a hybridization buffer. Subsequently, diluted and denatured probes were added to the pre-hybridized embryos followed by overnight incubation (68 °C) in a water bath. Post-hybridization washes were performed in increasing concentration of 2xSSC in the hybridization buffer. To reduce nonspecific signal, commercial blocking reagent (Roche) was used. Signal was visualized by overnight incubation with 1:5000 anti-DIG-AP antibody (Roche) at 4 °C followed by washing and addition of NBT and BCIP staining solution. After the staining was fully developed, staining solution was washed away and embryos were fixed in 4% PFA in 1 × PBS. For whole mount in situ imaging, embryos were mounted in glycerol and imaged on Nikon SMZ25 microscope. For each probe, WISH experiments were performed on at least 20 embryos obtained from at least three different breeding pairs.

## Supplementary Information

Below is the link to the electronic supplementary material.

Supplementary file1 (PDF 5145 KB)

Supplementary file2 (XLSX 13 KB)

Supplementary file3 (XLSX 1511 KB)

Supplementary file4 (XLSX 117 KB)

Supplementary file5 (XLSX 37 KB)

Supplementary file6 (XLSX 21 KB)

Supplementary file7 (XLSX 23 KB)

Supplementary file8 (XLSX 13 KB)

Supplementary file9 (XLSX 21 KB)

Supplementary file10 (XLSX 811 KB)

Supplementary file11 (XLSX 24 KB)

Supplementary file12 (XLSX 974 KB)

Supplementary file13 (XLSX 78 KB)

Supplementary file14 (XLSX 68 KB)

Supplementary file15 (XLSX 97 KB)

Supplementary file16 (XLSX 86 KB)

Supplementary file17 (XLSX 53 KB)

## Data Availability

All sequencing data have been deposited in the GEO database under accession number GSE160107.
