# Supplementary figures and images for: Genomic and physiological analyses of the zebrafish atrioventricular canal reveal molecular building blocks of the secondary pacemaker region
Source: Cell Mol Life Sci. 2021 Sep 23;78(19-20):6669–87. doi: 10.1007/s00018-021-03939-y (PMC8558220; doi:10.1007/s00018-021-03939-y)

**A**

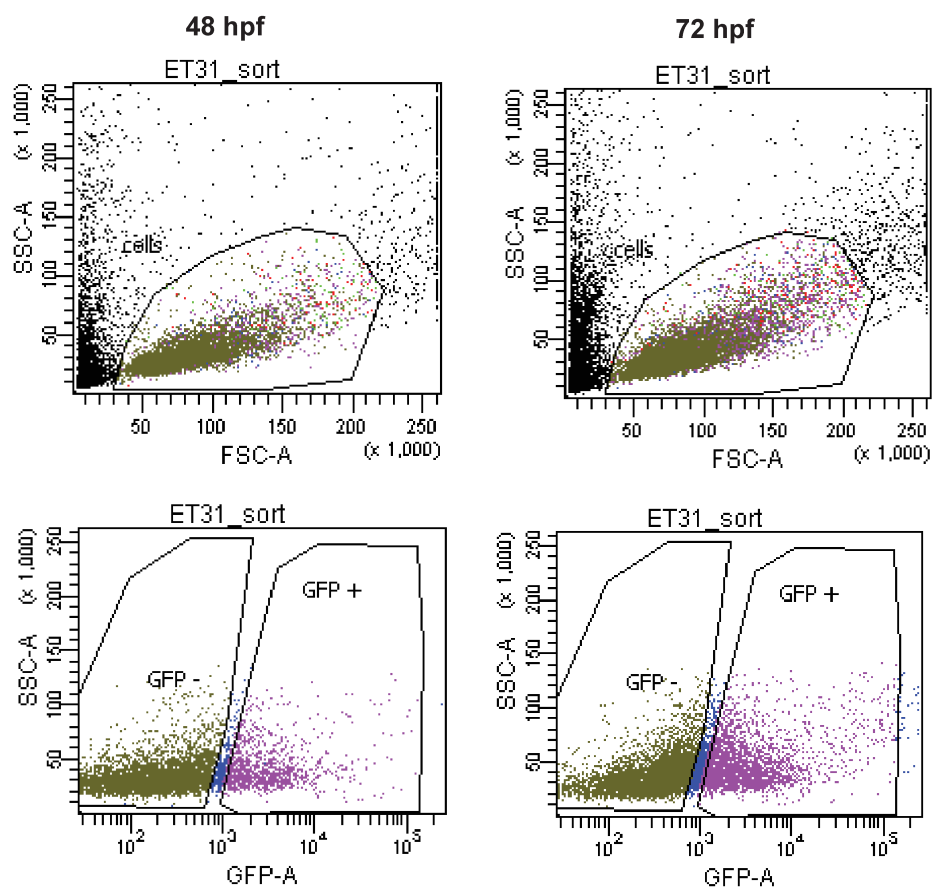

**B**

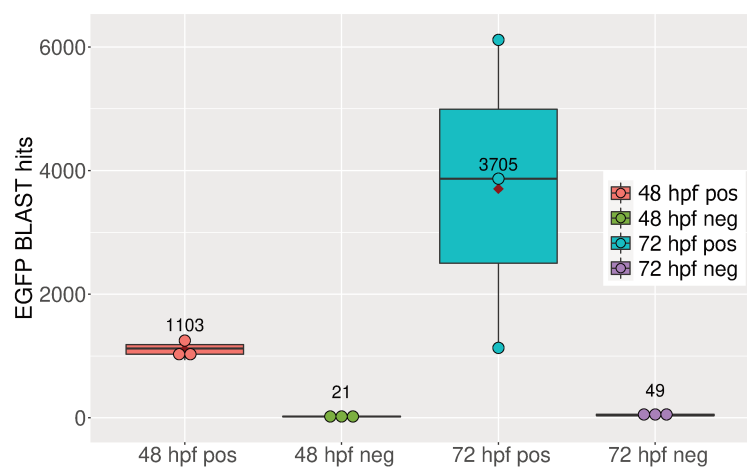

**C**

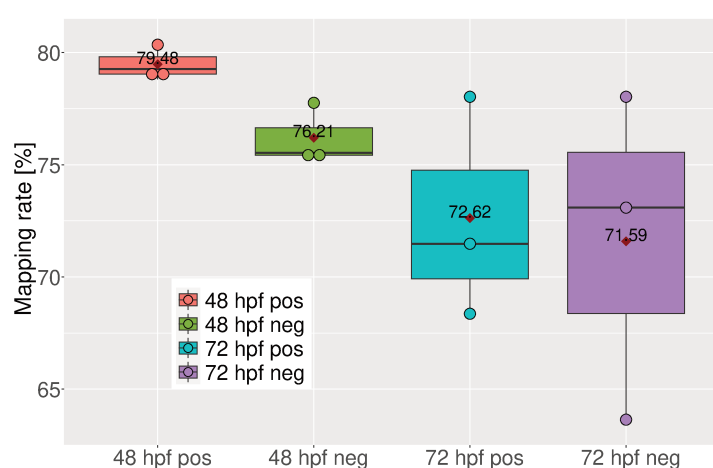

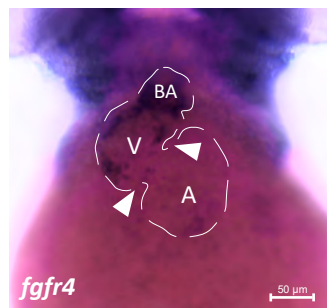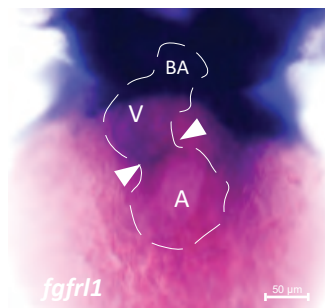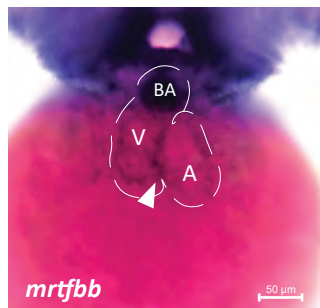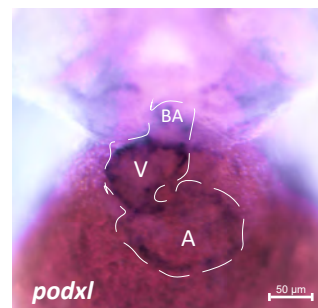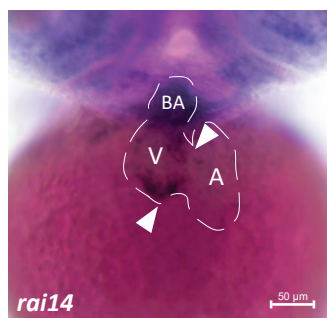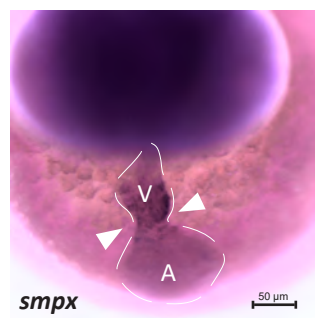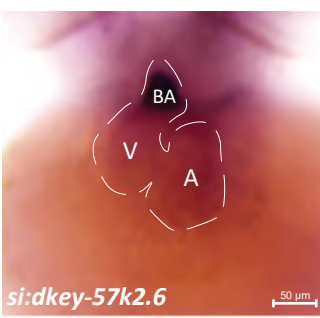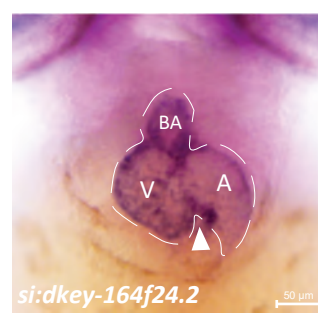

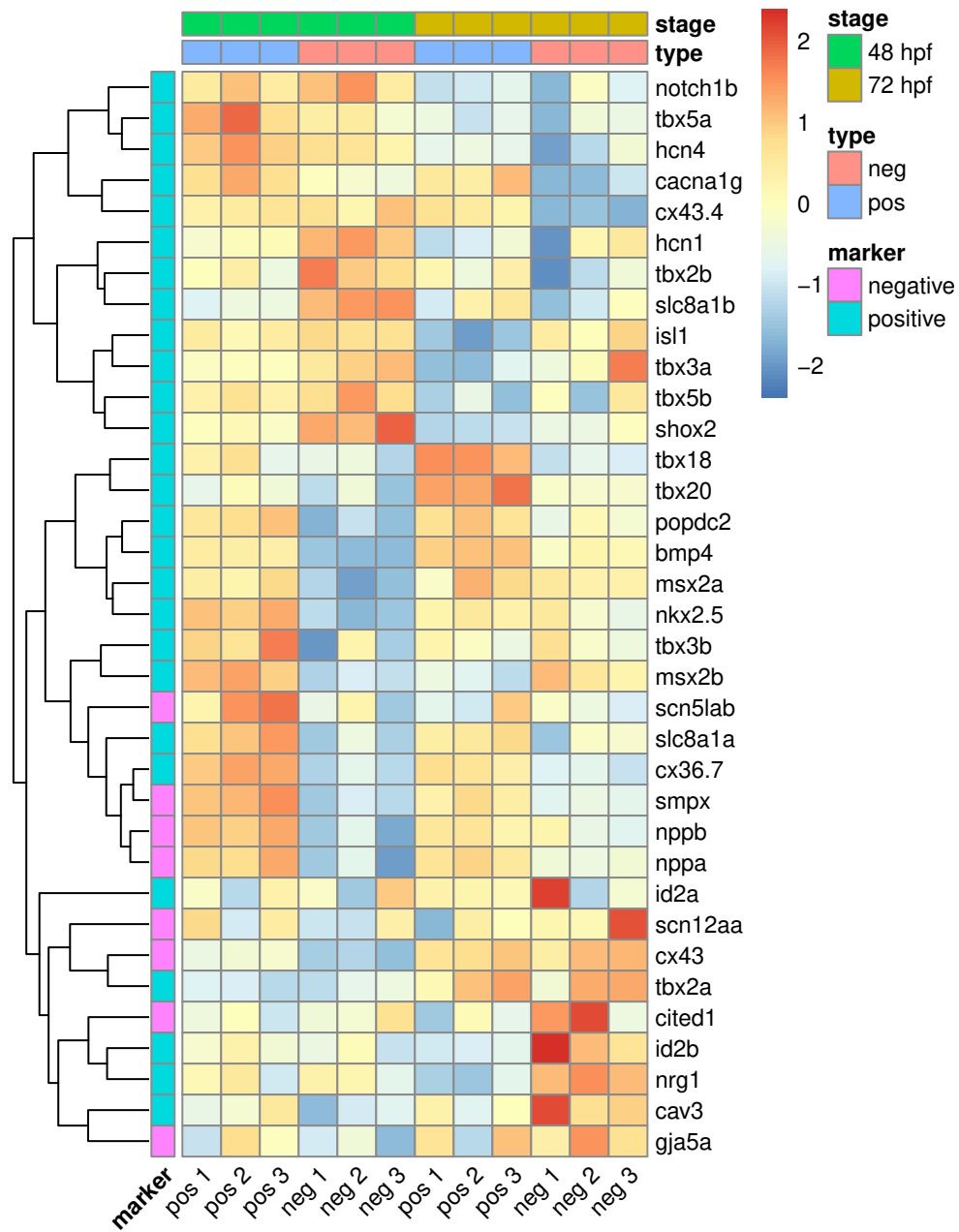

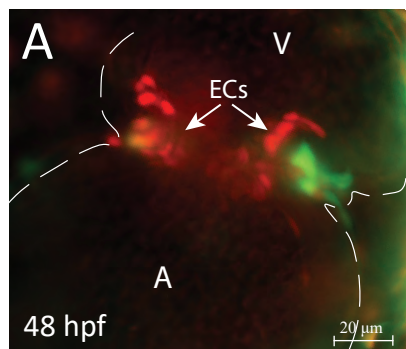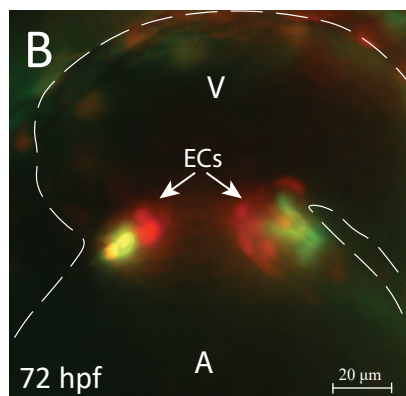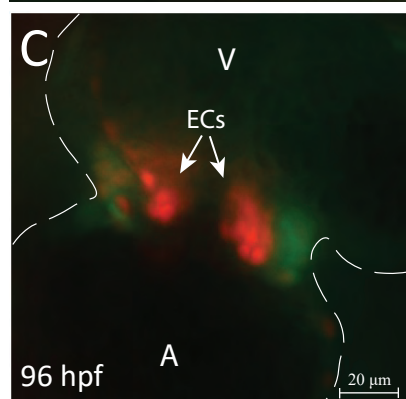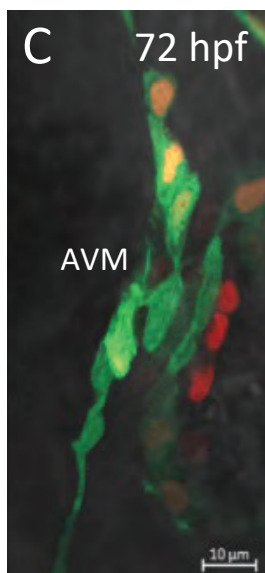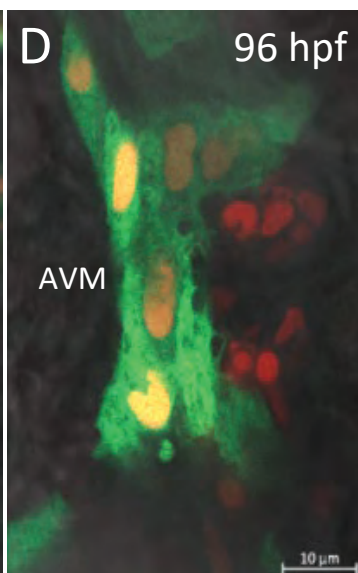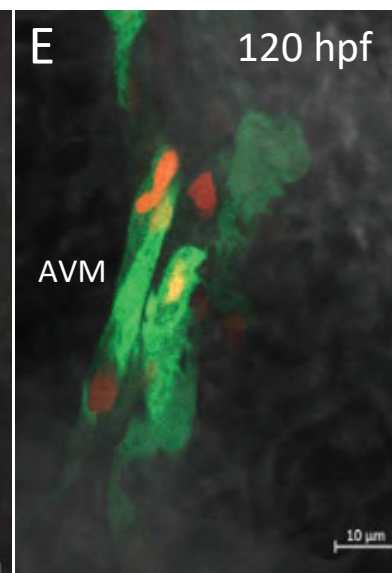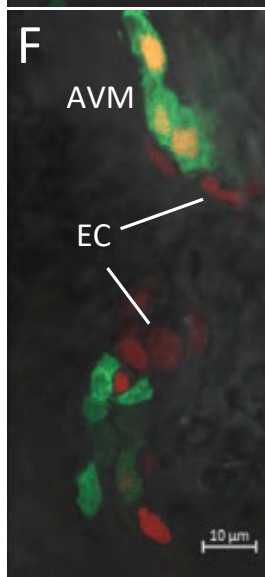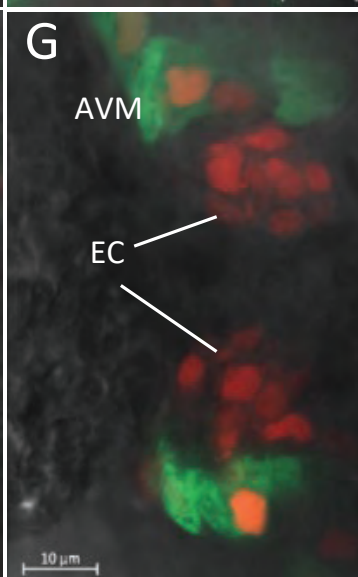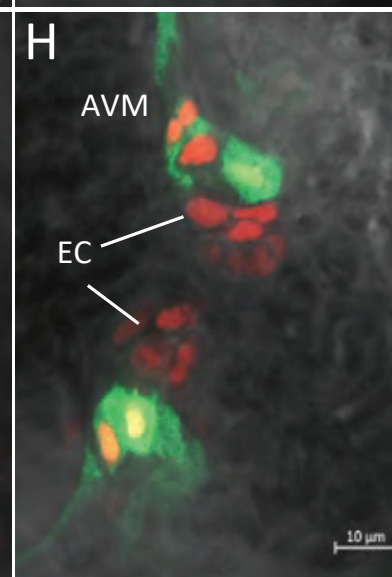

A

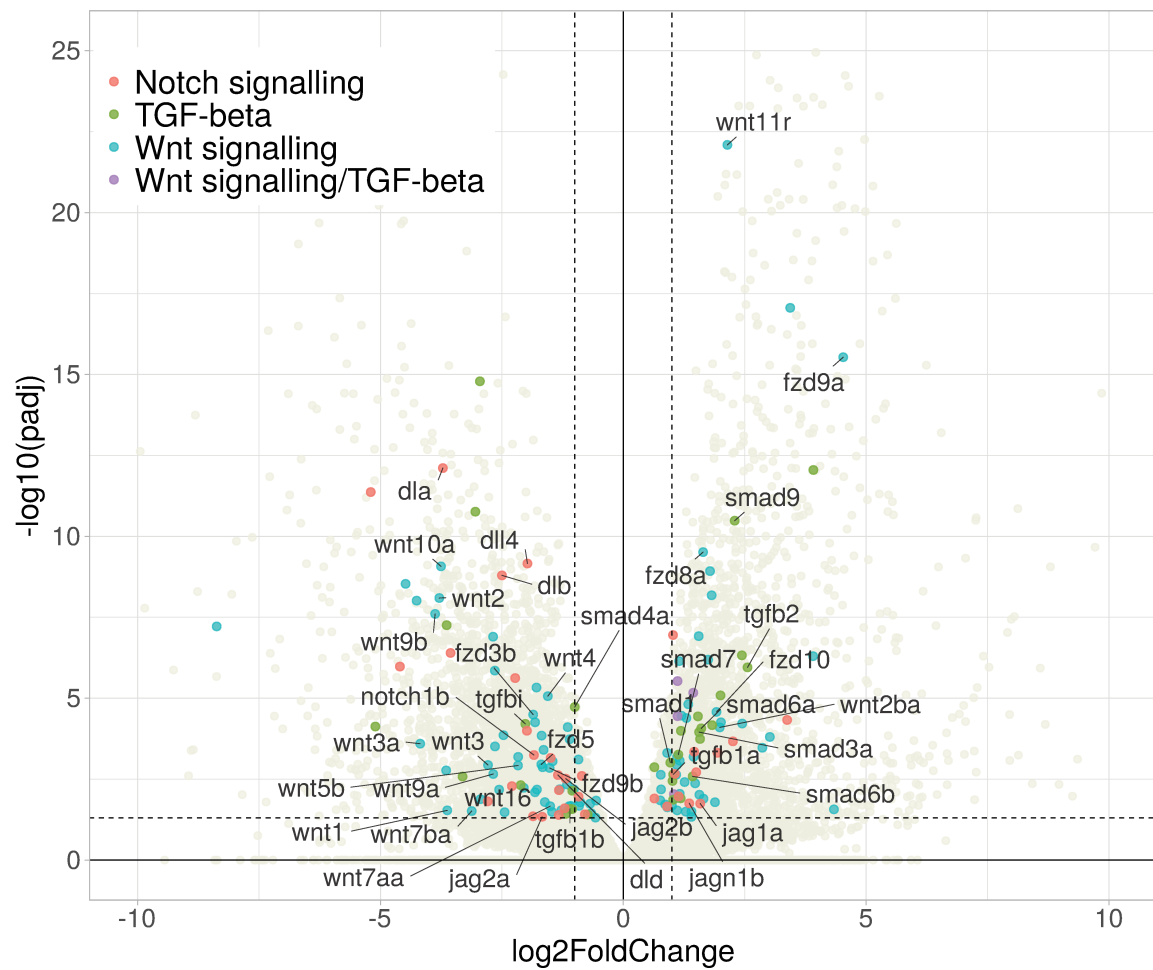

B

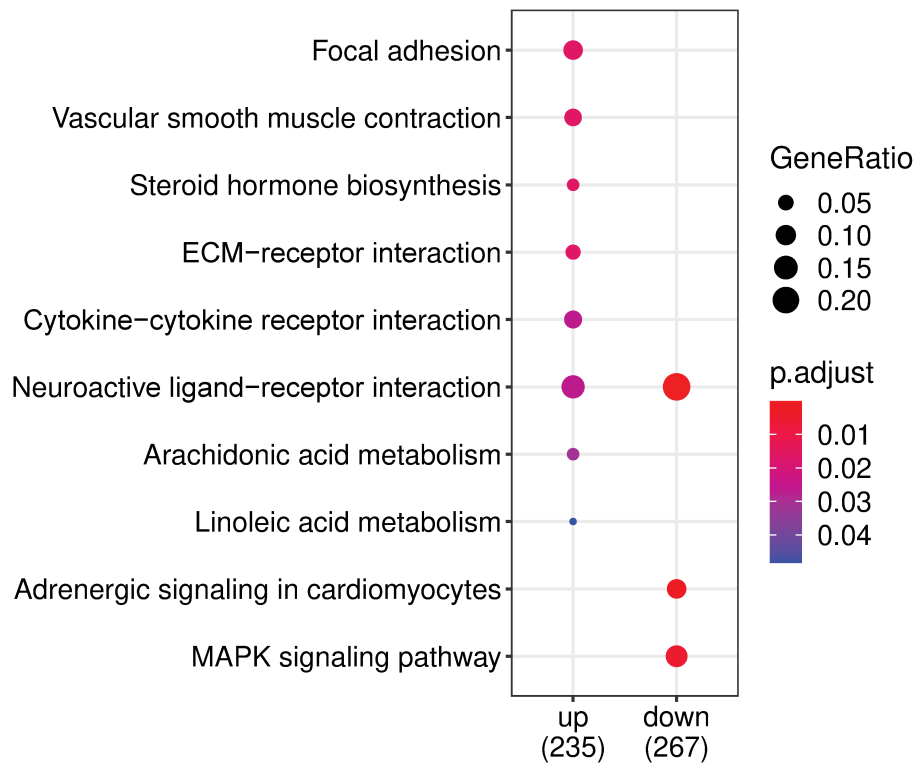

Supplement: Supplementary file 1 — Supplementary file1 (PDF 5145 KB) [file 18_2021_3939_MOESM1_ESM.pdf]
